# Integrated morphological, physiological, and transcriptomic analyses reveal the survival strategies of alpine *Rhododendron* under chronic heat stress

**DOI:** 10.3389/fpls.2026.1832629

**Published:** 2026-05-19

**Authors:** Mei Zhou, Huimin Li, Jianrui Kui, Fan Li, Shuang Zeng, Lu Zhang, Lvchun Peng, Weijia Xie, Shifeng Li, Chunlian Jin, Jie Song

**Affiliations:** 1 Flower Research Institute, Yunnan Academy of Agricultural Sciences, Key Lab of Yunnan Flower Breeding, National Engineering Research Center for Ornamental Horticulture, Kunming, China; 2 College of Landscape Architecture, Sichuan Agricultural University, Chengdu, China

**Keywords:** alpine *Rhododendron*, chronic heat stress, glutathione, membrane protection, transcriptomics, WGCNA

## Abstract

**Introduction:**

Alpine *Rhododendron* species possess high ornamental value, but their sensitivity to prolonged high temperatures limits their application in subtropical regions.

**Methods:**

To elucidate the mechanisms underlying chronic heat tolerance, distinct from classical acute heat-shock responses, this study integrated phenotypic screening, physiological profiling, transcriptomics, and biochemical analysis of the glutathione system.

**Results:**

The heat-tolerant cultivar 'Gommer Waterer' maintained membrane integrity under 30-day chronic heat stress despite accumulating substantially elevated H₂O₂ levels, a dissociation consistent with preferential detoxification of lipid peroxidation products rather than broad-spectrum ROS scavenging. Comparative time-course transcriptomics revealed that the tolerant cultivar mounted a substantially larger and earlier transcriptional response than the heat-sensitive cultivar 'Fenjingling', with sustained enrichment of phenylpropanoid, lipid metabolism, and glutathione pathways. Weighted gene co-expression network analysis identified a key module significantly correlated with heat resilience traits and enriched in glutathione metabolism. Biochemical assays corroborated these network findings, with the tolerant cultivar showing higher constitutive GPX activity, a stress-induced surge in GST activity, and active glutathione redox cycling, whereas the sensitive cultivar displayed elevated GSH/GSSG ratios accompanied by transcriptional repression of recycling enzymes *GR* and *DHAR*, suggesting redox stagnation rather than active defense.

**Discussion:**

Collectively, these findings suggest that glutathione-mediated detoxification of lipid peroxidation products is associated with membrane protection under prolonged thermal stress in this species, with the Theta-class hub gene *GSTT1* representing a candidate target for future functional validation and heat-tolerance breeding in woody ornamentals.

## 1 Introduction

Global warming has become an undeniable reality, with extreme heatwave events increasing in frequency, duration, and intensity worldwide (Lenton et al., 2023; Seth and Sebastian, 2024; He et al., 2025). *Rhododendron*, a flagship genus of the Ericaceae family comprising over 1,000 species, is renowned for its immense ornamental, medicinal, and ecological value (Rawat et al., 2024). However, most alpine *Rhododendron* species are highly thermosensitive, as they evolved in cool and high-altitude habitats with a narrow thermal niche and limited phenotypic plasticity, rendering them highly vulnerable under climate change (Li et al., 2023). Temperatures exceeding 35 °C—common in low-altitude urban landscapes—often trigger irreversible physiological damage, including leaf scorching, stunted growth, and high mortality rates (Shen et al., 2016; Zhang et al., 2021; Zhao et al., 2024; Zhang and Li, 2025). Despite decades of effort in heat-tolerance breeding, the low survival rate of alpine *Rhododendron* in subtropical summer conditions remains a major challenge for the global horticultural industry (Huyen et al., 2018; Liu et al., 2022).

The physiological responses of plants to heat stress (HS) involve a complex interplay of osmotic adjustment, antioxidant defense, and membrane stabilization (Sharma et al., 2024). HS triggers the overproduction of reactive oxygen species (ROS), inducing lipid peroxidation and compromising cellular membrane integrity. In response, resilient genotypes mobilize osmoprotectants (e.g., soluble sugars (SS) and proline (Pro)) to maintain cellular turgor, and activate antioxidant enzymes (e.g., SOD, POD) to neutralize ROS (Fathi et al., 2025; Su et al., 2019; Bao et al., 2024; Wang et al., 2024). Within this protective network, the non-enzymatic antioxidant glutathione (GSH) plays a central role in redox buffering during prolonged stress, protecting protein thiols from heat-induced denaturation and supporting sustained antioxidant function (Dorion et al., 2021; Hasanuzzaman et al., 2017). While these physiological markers have been extensively utilized to evaluate heat tolerance in various *Rhododendron* cultivars (Shen et al., 2016), they cannot resolve the regulatory networks governing long-term adaptation, necessitating transcriptomic approaches.

Unlike herbaceous models, perennial woody plants face unique challenges during prolonged summer heatwaves (Zhou et al., 2023). Plant responses to HS are generally categorized into acute heat-shock responses, occurring within hours to 3 days, and long-term thermal adaptation, extending to weeks or months (Mittler et al., 2012). Existing transcriptomic research on *Rhododendron* has predominantly focused on short-term induction, primarily involving the rapid activation of the heat shock transcription factor (HSF)-heat shock protein (HSP) pathway (Fang et al., 2017; Xu et al., 2022). However, chronic HS represents a more ecologically relevant condition: long-term survival under sustained HS requires profound structural remodeling, resource allocation trade-offs, and a progressive shift from temporary acclimation to long-term damage mitigation—processes that short-term studies cannot capture (Staacke et al., 2025). The molecular mechanisms underlying this transition remain particularly poorly understood in perennial woody ornamentals such as *Rhododendron*, where chronic thermal exposure represents a recurring ecological challenge (Liu et al., 2024; Xu et al., 2022).

In this study, we aimed to elucidate the mechanisms underlying long-term heat adaptation in alpine *Rhododendron*. We hypothesized that chronic HS induces distinct transcriptional and physiological reprogramming beyond classical acute heat-shock responses, specifically involving redox buffering and structural adaptation, and that differential survival between cultivars is driven by long-term metabolic reconfiguration rather than acute responses. To test this, we first screened 24 cultivars for thermotolerance and selected contrasting genotypes for a 30-day HS experiment. By integrating physiological analyzes with time-course transcriptomics, we sought to identify key regulatory networks and candidate genes associated with sustained heat tolerance.

## 2 Materials and methods

### 2.1 Plant materials and experimental site

A total of 24 representative *Rhododendron* cultivars were selected for this study (**Table 1**). Uniform seedlings (approximately 15 cm in height) were obtained from the Jinning Dachunhe Base of the Flower Research Institute, Yunnan Academy of Agricultural Sciences, China, and individually transplanted into plastic pots (120 mm diameter × 120 mm height). The cultivation substrate consisted of coarse peat (fiber length 5–20 mm, pH 5.0–5.5) and coarse coconut coir (fiber length 0–10 mm, EC ≤ 0.5 mS/cm, pH 5.5–6.0) mixed at a volume ratio of 5:3, supplemented with a 6-month controlled-release fertilizer (N:P:K = 16:8:12 + TE) at 3 g/L. Prior to experimental treatments, the seedlings were acclimated for three months in a greenhouse with 70% shading under standard horticultural management. Following acclimation, robust and disease-free seedlings with uniform growth were selected, with 12 independent plants maintained per cultivar for subsequent experiments.

**Table 1:** Cultivar name on 24 varieties of *Rhododendron*.

No	Cultivar name	No	Cultivar name
1	Prominence	13	Lord Roberts
2	Sweet Heart	14	Gommer Waterer
3	Yangchunxue	15	Fireworks
4	Hongzhuang	16	Fenjingling
5	Daiyu	17	Purple Dome
6	Huayan	18	Marian
7	Atlas	19	Lavender Prince
8	Nova Zembla	20	Strawberry
9	Sunfire	21	Red Monarch
10	Purple Rainbow	22	Kouzan
11	Wilgen’s Ruby	23	XXL
12	Roseum Elegans	24	Shinju-Hime

### 2.2 *In vitro* HS assay and heat injury evaluation

To rapidly and accurately evaluate the heat tolerance of the 24 *Rhododendron* cultivars, an *in vitro* HS assay was conducted using detached leaves. This method focuses on cellular-level thermotolerance by excluding whole-plant physiological variables. This approach is well-established in woody plant thermotolerance research, as cellular membrane stability under acute thermal stress has been shown to strongly correlate with *in vivo* field resilience (Zhao et al., 2010; Shen et al., 2016). A temperature of 45°C was applied as a discriminating threshold to induce rapid and phenotypically divergent responses among cultivars within a short treatment window.

Healthy, mature functional leaves were collected from the upper-middle branches of the acclimated seedlings. For each cultivar, three independent biological replicates were established, with each replicate consisting of 12 leaves collected individually from each of the 12 stock plants (one leaf per plant), yielding 36 leaves per cultivar in total. Leaves were excised at the petiole, temporarily stored in polyethylene bags with moist filter paper to maintain turgor, and immediately transported to the laboratory.

To prevent dehydration during treatment, the leaf petioles were inserted into water-saturated sponges within Petri dishes. The dishes were covered with plastic wrap (with small perforations) to minimize evaporation while allowing gas exchange. Subsequently, the dishes were placed in a controlled environmental chamber (SPX-250B-G) and subjected to severe HS at 45 °C. The chamber conditions were set at a 12/12 h (light/dark) photoperiod, a light intensity of 1800 lx, and 75% relative humidity. Phenotypic changes were continuously monitored, and photographic records were taken at 24, 36, 48, and 60 h post-treatment. Control leaves were maintained at 25 °C under identical light and humidity conditions.

Based on the morphological deterioration (e.g., wilting, browning, and necrosis), the Heat Injury index (HI) was evaluated on a scale of 0 to 5 (**Table 2**), following modified protocols from previous studies (Zhao et al., 2010; Shen et al., 2016). The HI for each cultivar was calculated as follows:

**Table 2:** Classification standards of the heat injury (HI) index for *Rhododendron* leaves.

Index level	Leaf symptoms/phenotypic description	Reference image
Level 0	The leaf color is normal; no symptoms of heat injury are observed.	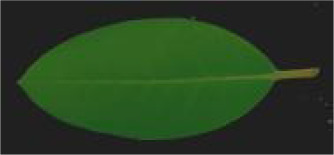
Level 1	The leaf turns yellowish-green and exhibits curling; chlorosis or wilting begins at the leaf margins.	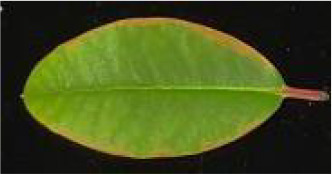
Level 2	A few necrotic spots appear on the leaf; approximately 1/4 to 1/3 of the leaf area is chlorotic or wilted.	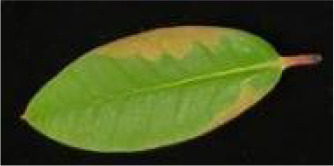
Level 3	Approximately 1/3 to 1/2 of the leaf area is yellowed or wilted.	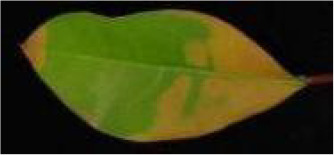
Level 4	More than 1/2 of the leaf area is severely wilted and yellowed.	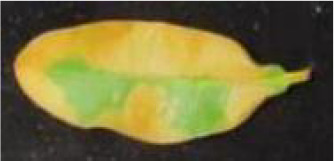
Level 5	The leaf is entirely yellowed, desiccated, or completely wilted.	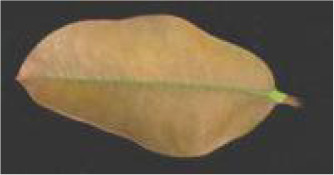


$$\text{HI}=\frac{\sum (ni\times vi)}{N\times V}\times 100$$

Where *vi* represents the specific assigned heat injury level (0 to 5), *ni* is the number of leaves exhibiting symptoms at level *vi*, *N* is the total number of leaves evaluated per cultivar, and *V* is the maximum possible injury level (5).

### 2.3 Chronic HS treatment and sampling

Based on the preliminary *in vitro* heat injury evaluation, five representative *Rhododendron* cultivars spanning contrasting levels of heat tolerance were selected for subsequent physiological analyzes: the highly tolerant ‘Gommer Waterer’ (GW) and ‘Atlas’, the moderately tolerant ‘Yangchunxue’, and the heat-sensitive ‘Shinju-hime’ and ‘Huayan’. Selection was based on hierarchical clustering of HI values, with cultivars chosen to represent each distinct tolerance tier identified in the screening. The acclimated potted seedlings (five pots per cultivar) were subsequently transferred to a controlled environmental chamber.

To accurately simulate the natural diurnal high-temperature dynamics of a subtropical summer, the chamber was programmed with a 24-h fluctuating temperature cycle based on typical subtropical summer climate profiles observed in Chengdu, Sichuan Province, China. The thermal regime was set as follows: 24 °C for 5 h (0:00–5:00, dark), 28 °C for 3 h (5:00–8:00, light), a peak of 35 °C for 8 h (8:00–16:00, light), 32 °C for 3 h (16:00–19:00, light), and 28 °C for 5 h (19:00–24:00, dark). The photoperiod was maintained at 14/10 h (light/dark) with a daytime light intensity of 1800 lx, a constant relative humidity of 75%, and a gentle internal chamber airflow of 0.5 m/s. To eliminate the confounding effects of heat-induced drought stress, the cultivation substrate was regularly supplemented with water. Seedlings maintained at a constant 24 °C for 7 days served as the baseline control (0 d).

To capture the dynamic physiological and molecular responses, mature functional leaves from uniform canopy positions were sampled at 0, 5, 10, 15, 20, 25, and 30 days post-treatment. To ensure temporal and physiological consistency, all samplings were strictly executed at the end of the daily 35°C peak stress period (at 16:00). At each time point, three independent biological replicates were sampled per cultivar. To ensure robust representation and minimize individual variance, each biological replicate was generated by pooling functional leaves collected from the five distinct plants assigned to that specific replicate group. Leaves were rapidly wiped clean, wrapped in aluminum foil, immediately snap-frozen in liquid nitrogen, and stored at −80 °C for subsequent assays.

### 2.4 Physiological indices

The sampled leaves were immediately snap-frozen in liquid nitrogen and stored at −80 °C. For all subsequent physiological assays, approximately 0.1 g of frozen leaf tissue was used per extraction. The physiological indices were determined as follows:

Osmoregulation substances: Free proline (Pro) and soluble sugar (SS) contents were quantified using the acidic ninhydrin method (Bates et al., 1973) and anthrone colorimetry (Buysse and Merckx, 1993), respectively. Soluble protein (SP) content was determined via the Coomassie Brilliant Blue G-250 staining method (Bradford, 1976).

Oxidative damage markers: The accumulation of hydrogen peroxide (H_2_O_2_) was analyzed using the titanium sulfate colorimetric assay (Li et al., 2010). Lipid peroxidation was evaluated by measuring malondialdehyde (MDA) content via the thiobarbituric acid (TBA) reaction method (Jiang et al., 2022).

Antioxidant enzyme activities: The activities of superoxide dismutase (SOD), peroxidase (POD), and catalase (CAT) were determined using corresponding commercial assay kits (YX-W-A500-WST-8, YX-W-A502, and YX-W-A501; Shanghai Youxuan Biological Technology, China) strictly following the manufacturer’s instructions.

### 2.5 Transcriptomic sequencing and bioinformatics analysis

#### 2.5.1 RNA extraction, transcriptome sequencing, and data analysis

**Figure 1:**
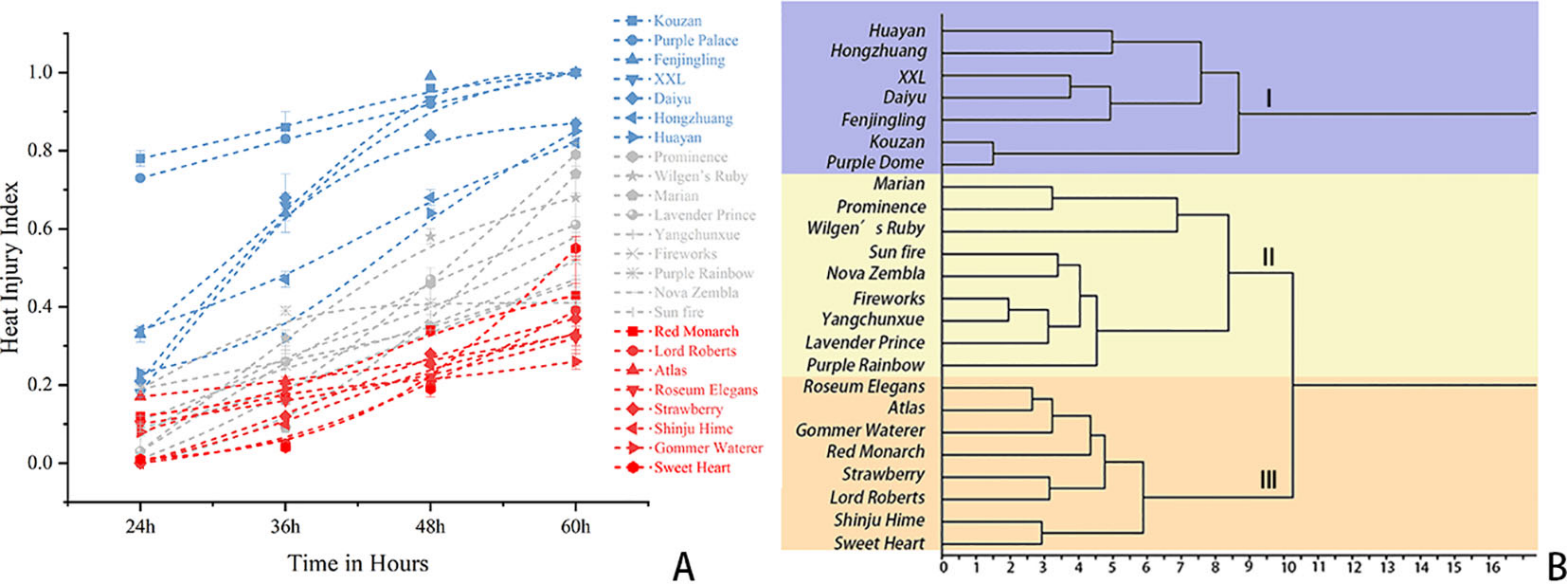
Phenotypic response and hierarchical clustering of 24 *Rhododendron* cultivars under HS. **(A)** Dynamic progression of leaf heat injury index (HI) under 45 °C stress. The line graph highlights contrasting injury kinetics: sensitive cultivars (blue spectrum) exhibit rapid, early-onset injury escalation, while tolerant cultivars (red spectrum) demonstrate a prolonged maintenance phase. **(B)** Hierarchical clustering dendrogram based on thermal resilience. The dendrogram classifies the germplasm into three tiers: Tier I (Sensitive, purple), Tier II (Moderately Tolerant, light purple), and Tier III (Highly Tolerant, orange) based on phenotypic damage trajectories across 24 h, 36 h, 48 h, and 60 h.

Total RNA was extracted from frozen leaf samples of ‘GW’ and ‘FJL’ cultivars using the Universal Plant Total RNA Extraction Kit (Vazyme, Nanjing, China) (Zhou et al., 2025). ‘FJL’ was selected as the heat-sensitive contrast based on its rapid injury progression during the initial phenotypic screening (**Figure 1B**). RNA integrity and concentration were assessed using an Agilent 2100 Bioanalyzer (Agilent Technologies, USA) and a NanoPhotometer (IMPLEN, CA, USA). mRNA was enriched using oligo(dT) beads and fragmented to construct cDNA libraries with the Hieff NGS^®^ DNA Selection Beads (Yeasen, Shanghai, China). Sequencing was performed on the Illumina NovaSeq 6000 platform by Biomarker Technologies (Beijing, China). Three independent biological replicates were used per cultivar per time point, yielding a total of 30 libraries. Each biological replicate consisted of pooled functional leaves collected from the five distinct plants assigned to that specific replicate group, sampled strictly at the end of the daily 35°C peak stress period to ensure temporal and physiological consistency across all replicates.

Raw reads were processed using fastp (v0.18.0) (Chen et al., 2018) to remove adapters and low-quality data. The sequencing yielded 150-bp paired-end (PE150) reads, generating a minimum of 35 million clean reads per library (30 libraries in total), with Q30 values consistently exceeding 96%. The resulting clean reads were aligned to the reference genome of *Rhododendron delavayi* (GigaDB accession: 100331) (Zhang et al., 2017) using HISAT2 (v2.1.0) (Kim et al., 2019) with default parameters. The overall mapping rate across all 30 libraries ranged from 78.3% to 85.6%, with a mean mapping rate of 82.4%. The relatively moderate mapping efficiency is attributable to the inherent genomic divergence between the sequenced cultivars and the *R. delavayi* reference genome. To mitigate this limitation, stringent alignment quality thresholds were applied throughout the analysis to ensure accurate transcript quantification. Transcripts were reconstructed using StringTie (v1.3.4 (Pertea et al., 2015)), and gene expression levels were normalized as fragments per kilobase million (FPKM) using RNA-Seq by Expectation-Maximization (RSEM) (Li and Dewey, 2011). Differentially expressed genes (DEGs) were identified via DESeq2 (v1.22.2) (Love et al., 2014) with thresholds of |log_2(Fold Change)|≥1 and false discovery rate (FDR) < 0.05, with multiple testing correction applied using the Benjamini-Hochberg procedure (Benjamini and Hochberg, 1995) to control the false discovery rate across all pairwise comparisons.

#### 2.5.2 Weighted gene co-expression network analysis

Weighted Gene Co-expression Network Analysis (WGCNA, v1.66) was performed on 17,340 genes passing variance filtering to identify modules associated with HS. A signed network was constructed with a soft-thresholding power of *β* = 12, which achieved a scale-free topology fit index (R^2^) of 0.855. Modules were detected using the dynamic tree-cut method (minModuleSize = 30) and merged with a dissimilarity threshold of 0.25, yielding 18 co-expression modules.

To evaluate module-trait relationships and prevent oversimplification of network biology, Pearson correlation analysis was performed between module eigengenes (MEs) and multiple physiological traits (SP, MDA, POD, and H_2_O_2_), measured at 0, 5, 10, 20, and 30 d post-treatment. To account for innate baseline differences among cultivars, the net heat-induced change in MDA (ΔMDA=MDA_treatment_-MDA_0d_) was utilized as the primary marker for terminal membrane damage. Hub genes were defined based on Module Membership (MM) values, with a threshold of MM > 0.90 applied to select high-confidence candidates. The co-expression network was visualized using Cytoscape (v3.10.3) with an edge weight threshold of 0.15.

### 2.6 Validation of DEGs by qRT-PCR

Quantitative real-time PCR (qRT-PCR) was performed to validate the expression levels of nine glutathione (GSH)-related genes identified from the co-expression network. Total RNA was reverse-transcribed into cDNA using the HiScript^®^ III RT SuperMix for qPCR (+gDNA wiper) (Vazyme, Nanjing, China). The qRT-PCR reactions were conducted on a CFX96 Real-Time PCR Detection System (Bio-Rad, Hercules, CA, United States) using ChamQ SYBR Color qPCR Master Mix (Vazyme). The *EF1-α* gene was employed as the internal control for normalization, as our preliminary screening of multiple candidate genes (including *Actin*) demonstrated it to be the most consistently expressed across all heat treatments, a finding consistent with previous validations in *Rhododendron* (Zhang et al., 2022). Primers were designed using Primer Premier 6.0 (Palo Alto, CA, United States), and their specific sequences are listed in **Supplementary Table 1**. Relative expression levels were calculated using the 2^-ΔΔCt^ method (Livak and Schmittgen, 2001).

### 2.7 Determination of glutathione-related antioxidant system

The activities of glutathione peroxidase (GPX) and glutathione S-transferase (GST), alongside the concentrations of reduced glutathione (GSH) and oxidized glutathione (GSSG), were quantified using commercial biochemical assay kits (Sangon Biotech Co., Ltd., Shanghai, China) via UV-Vis spectrophotometry. The assays were performed according to the manufacturer’s instructions.

Briefly, approximately 0.1 g of frozen leaf tissue was homogenized in 1 mL of pre-chilled specific extraction buffer provided in each kit. After centrifugation at 8,000 × *g* for 10 min at 4°C, the supernatant was collected for immediate enzymatic and biochemical analyzes.

GPX activity (Kit No. D799612) was measured based on its ability to catalyze the oxidation of GSH by H_2_O_2_. The residual GSH was then quantified by reacting with 5,5’-dithiobis-(2-nitrobenzoic acid) (DTNB), and the decrease in absorbance was recorded at 412 nm. One unit (U) of GPX activity was defined as the amount of enzyme that catalyzes the oxidation of 1 μmol of GSH per minute per gram of fresh weight (*U·g*^-1^ FW).

GST activity (Kit No. D799613) was determined by continuously monitoring the formation of the GS-DNB conjugate—a product of the reaction between GSH and the substrate 1-chloro-2,4-dinitrobenzene (CDNB). The increase in absorbance was recorded at 340 nm. One unit (U) of GST activity was defined as the amount of enzyme required to conjugate 1 μmol of CDNB with GSH per minute per gram of fresh weight (*U·g*^-1^ FW).

GSH and GSSG contents were determined separately using their respective kits (GSH: Kit No. D799614; GSSG: Kit No. D799616) via the DTNB-based colorimetric method. For total GSH content, the reduction of DTNB was directly measured at 412 nm. In a parallel assay for GSSG quantification, endogenous GSH in the extract was specifically masked using 2-vinylpyridine prior to the DTNB reaction, and the absorbance was similarly read at 412 nm. The actual reduced GSH content was calculated by subtracting twice the measured GSSG value from the total glutathione pool. Results are expressed as μmol/g FW, and the cellular redox status was evaluated by calculating the GSH/GSSG ratio.

### 2.8 Statistical analysis

Statistical analyzes of the leaf physiological data were performed using SPSS Statistics 26.0 (IBM, Armonk, NY, United States). Prior to analysis, data were tested for normality using the Shapiro-Wilk test and for homogeneity of variance using Levene’s test. Analyzes of variance (ANOVA) and Duncan’s multiple range test were employed to conduct robust pairwise comparisons among different time points and cultivars, with significance defined at *P* < 0.05. Data standardization and principal component analysis (PCA) were conducted using Origin 2021 (OriginLab, Northampton, MA, United States) (Jiang et al., 2022). The processing, statistical analysis, and visualization of qRT-PCR data, as well as its correlation with RNA-Seq results, were performed using GraphPad Prism 10.0 (GraphPad Software, San Diego, CA, United States). All experimental data are expressed as the mean ± standard deviation (SD) of at least three biological replicates.

## 3 Results

### 3.1 Phenotypic evaluation and heat injury classification of 24 cultivars

The heat injury (HI) index of 24 representative *Rhododendron* cultivars was measured under 45 °C stress over 60 h (**Table 3**). HI values increased across all cultivars over time, with variation in the rate of increase among genotypes (**Figure 1A**). Phenotypic divergence among the population became evident within the first 24 h of stress. Cultivars such as ‘Kouzan’ and ‘Purple Dome’ exhibited HI values of 0.78 ± 0.02 and 0.73 ± 0.00, respectively, at 24 h. In contrast, cultivars including ‘Lord Roberts’, ‘Marian’, ‘Strawberry’, and ‘Shinju-hime’ maintained HI values of 0.00 during this period. Between 24 h and 60 h, the HI of genotypes such as ‘Kouzan’, ‘Purple Dome’, and ‘XXL’ approached the maximum value of 1.00. Conversely, ‘Gommer Waterer’ and ‘Roseum Elegans’ maintained lower HI values throughout the 60 h treatment. Hierarchical cluster analysis was performed using the HI dataset across all time points (**Figure 1B**).

**Table 3:** Heat injury (HI) index of 24 *Rhododendron* cultivars under HS (45 °C) at different durations.

No.	Cultivar name	(hi)24h	(hi)36h	(hi)48h	(hi)60h
1	Prominence	0.01 ± 0.01lm	0.16 ± 0.01jk	0.46 ± 0.04h	0.79 ± 0.03cd
2	Sweetheart	0.01 ± 0.01lm	0.04 ± 0.01m	0.19 ± 0.02o	0.55 ± 0.03fg
3	Yangchunxue	0.07 ± 0.05k	0.29 ± 0.02fg	0.38 ± 0.00j	0.58 ± 0.01fg
4	Hongzhuang	0.34 ± 0.01c	0.47 ± 0.02d	0.68 ± 0.02e	0.82 ± 0.01bc
5	Daiyu	0.21 ± 0.01de	0.68 ± 0.06b	0.84 ± 0.01d	0.87 ± 0.01b
6	Huayan	0.23 ± 0.01d	0.32 ± 0.02f	0.64 ± 0.01f	0.85 ± 0.02bc
7	Atlas	0.17 ± 0.00g	0.21 ± 0.01i	0.26 ± 0.01m	0.33 ± 0.03k
8	Nova Zembla	0.19 ± 0.01ef	0.26 ± 0.01	0.34 ± 0.01k	0.47 ± 0.04hi
9	Sunfire	0.09 ± 0.01ijk	0.18 ± 0.00ijk	0.34 ± 0.01k	0.46 ± 0.02hi
10	Purple Rainbow	0.19 ± 0.01efg	0.39 ± 0.01e	0.41 ± 0.01i	0.41 ± 0.06ij
11	Wilgen’s Ruby	0.03 ± 0.00l	0.32 ± 0.02f	0.58 ± 0.02g	0.68 ± 0.06e
12	Roseum Elegans	0.10 ± 0.00hij	0.16 ± 0.01k	0.22 ± 0.01n	0.32 ± 0.03kl
13	Lord Roberts	0.00 ± 0.00m	0.05 ± 0.00m	0.20 ± 0.00o	0.39 ± 0.14jk
14	Gommer Waterer	0.08 ± 0.00jk	0.19 ± 0.01ij	0.21 ± 0.01no	0.26 ± 0.02l
15	Fireworks	0.11 ± 0.02hi	0.25 ± 0.03h	0.34 ± 0.01k	0.52 ± 0.07gh
16	Fenjingling	0.33 ± 0.02c	0.64 ± 0.05c	0.99 ± 0.00a	1.00 ± 0.00a
17	Purple Dome	0.73 ± 0.00b	0.83 ± 0.00a	0.92 ± 0.01c	1.00 ± 0.00a
18	Marian	0.00 ± 0.00m	0.09 ± 0.00l	0.35 ± 0.01k	0.74 ± 0.05de
19	Lavender Prince	0.03 ± 0.00l	0.26 ± 0.01gh	0.47 ± 0.00h	0.61 ± 0.02f
20	Strawberry	0.00 ± 0.00m	0.12 ± 0.00l	0.28 ± 0.00l	0.37 ± 0.05jk
21	Red Monarch	0.12 ± 0.01h	0.17 ± 0.00jk	0.34 ± 0.01k	0.43 ± 0.03ij
22	Kouzan	0.78 ± 0.02a	0.86 ± 0.04a	0.96 ± 0.01b	1.00 ± 0.00a
23	XXL	0.18 ± 0.01fg	0.66 ± 0.01bc	0.93 ± 0.01c	1.00 ± 0.00a
24	Shinju-hime	0.00 ± 0.00m	0.10 ± 0.01l	0.25 ± 0.00m	0.33 ± 0.03k

Values are expressed as mean ± standard deviation (n=3). Different lowercase letters within the same column indicate significant differences between cultivars at *P <* 0.05 according to Duncan’s test.

**Figure 2:**
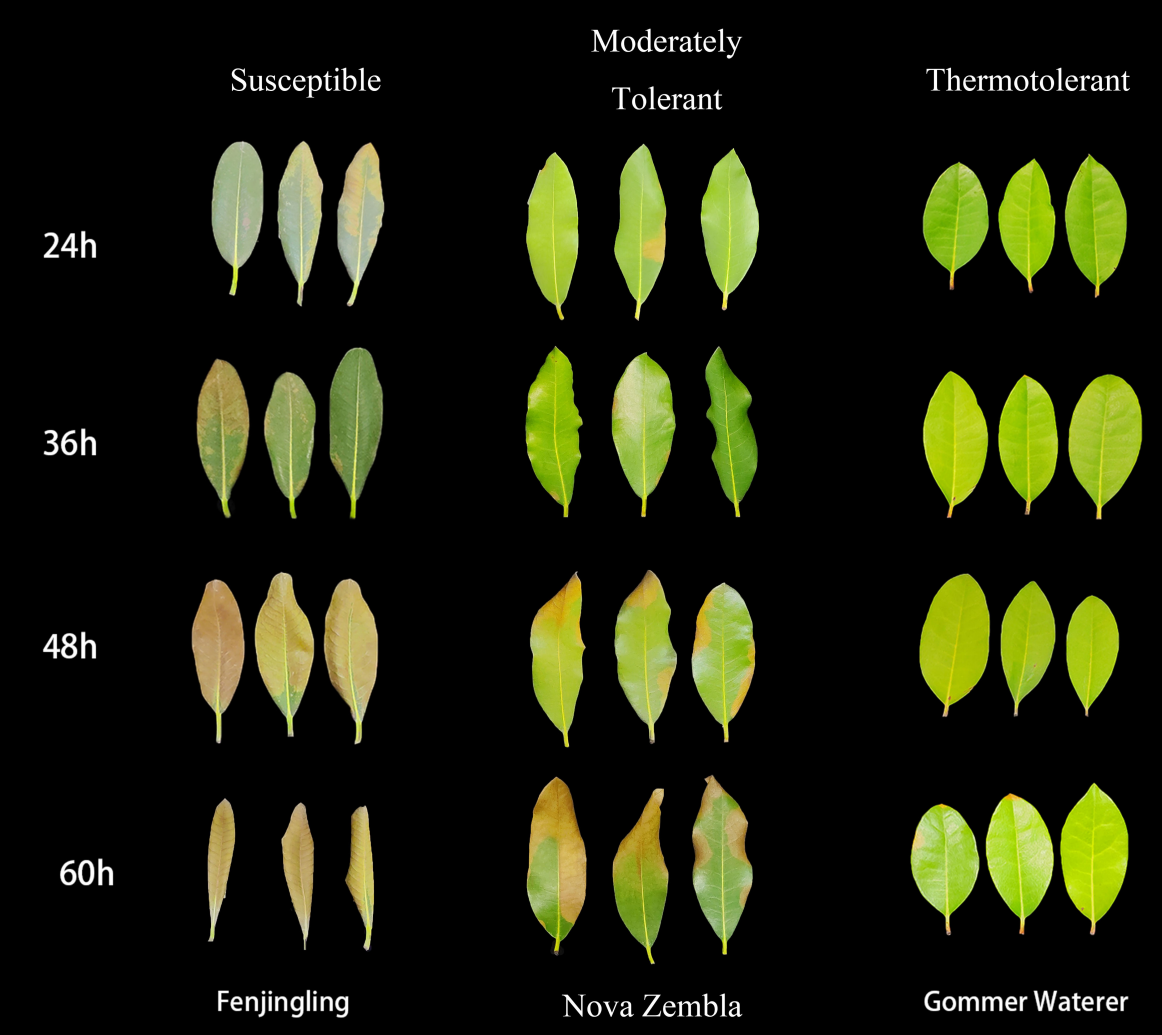
Morphological responses of representative *Rhododendron* cultivars to HS. Detached leaves from three cultivars with distinct thermotolerance—’Fenjingling’ (susceptible), ‘Nova Zembla’ (moderately tolerant), and ‘Gommer Waterer’ (thermotolerant)—are shown at 24, 36, 48, and 60 h of 45 °C exposure.

The representative phenotypic characteristics of the resulting clusters are illustrated in **Figure 2**, using ‘Fenjingling’ (susceptible), ‘Nova Zembla’ (moderately tolerant), and ‘Gommer Waterer’ (thermotolerant) as typical examples. The 24 cultivars were categorized into three groups. The first category (susceptible) comprised 7 cultivars, including ‘Fenjingling’, ‘Purple Dome’, ‘Kouzan’, and ‘XXL’. Leaves of these cultivars exhibited complete wilting and desiccation by 60 h. The second category (moderately tolerant) consisted of 9 cultivars, such as ‘Prominence’, ‘Marian’, and ‘Wilgen’s Ruby’. These cultivars developed necrotic spots and marginal browning after 36 h. The third category (thermotolerant) included 8 cultivars, including ‘Gommer Waterer’ and ‘Roseum Elegans’, which maintained their overall morphological stability, exhibiting only mild heat injury symptoms by 60 h.

### 3.2 Physiological response mechanisms and heat tolerance evaluation

**Figure 3:**
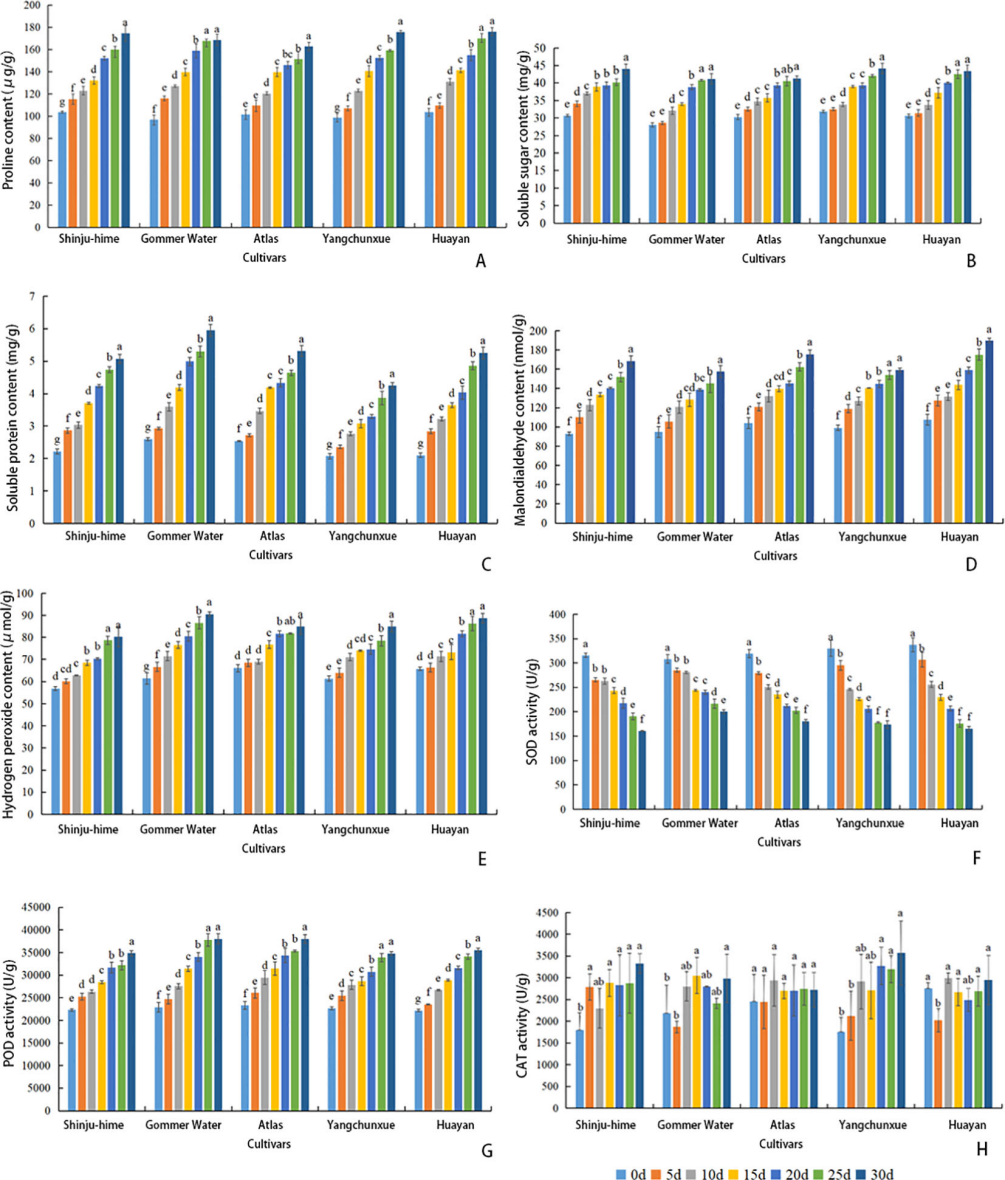
Dynamics of physiological parameters in five *Rhododendron* cultivars under chronic HS. Data are expressed as mean ± SD (n = 3). Different lowercase letters indicate significant differences between time points within each cultivar at (Duncan’s test). **(A)** Proline; **(B)** Soluble sugar; **(C)** Soluble protein; **(D)** MDA; **(E)** H_2_O_2_; **(F)** SOD; **(G)** POD; **(H)** CAT.

To evaluate the cellular responses of the five *Rhododendron* cultivars to chronic HS, we determined the activities of POD, SOD, and CAT, as well as the contents of MDA, H_2_O_2_, Pro, SS, and SP in leaves (**Figure 3**). Between 0 and 30 d, the contents of Pro, SS, SP, H_2_O_2_, and MDA, as well as POD activity, increased across all five cultivars, reaching their maximum values at 30 d. ‘Gommer Waterer’ maintained relatively higher levels of SP and POD activity compared to most cultivars, while ‘Yangchunxue’ showed the highest accumulation of Pro. Conversely, SOD activity displayed a universal declining trend as HS intensified; however, ‘Gommer Waterer’ retained higher residual SOD activity than the sensitive ‘Huayan’ at 30 d. CAT activity exhibited irregular fluctuations across genotypes without a discernible trend.

‘Shinju-hime’ and ‘Huayan’ experienced a greater increase in MDA concentration than other cultivars. In contrast, ‘Gommer Waterer’ and ‘Yangchunxue’ maintained relatively lower MDA accumulation. Notably, while ‘Gommer Waterer’ recorded the highest H_2_O_2_ content at 30 d, its MDA level remained comparatively stable. Furthermore, unlike the continuous escalation observed in other genotypes, the accumulation of Pro and SS in ‘Gommer Waterer’ reached a statistical plateau during the late stage of stress (25–30 d), showing no significant difference between these two time points (**Figures 3A, B**).

**Figure 4:**
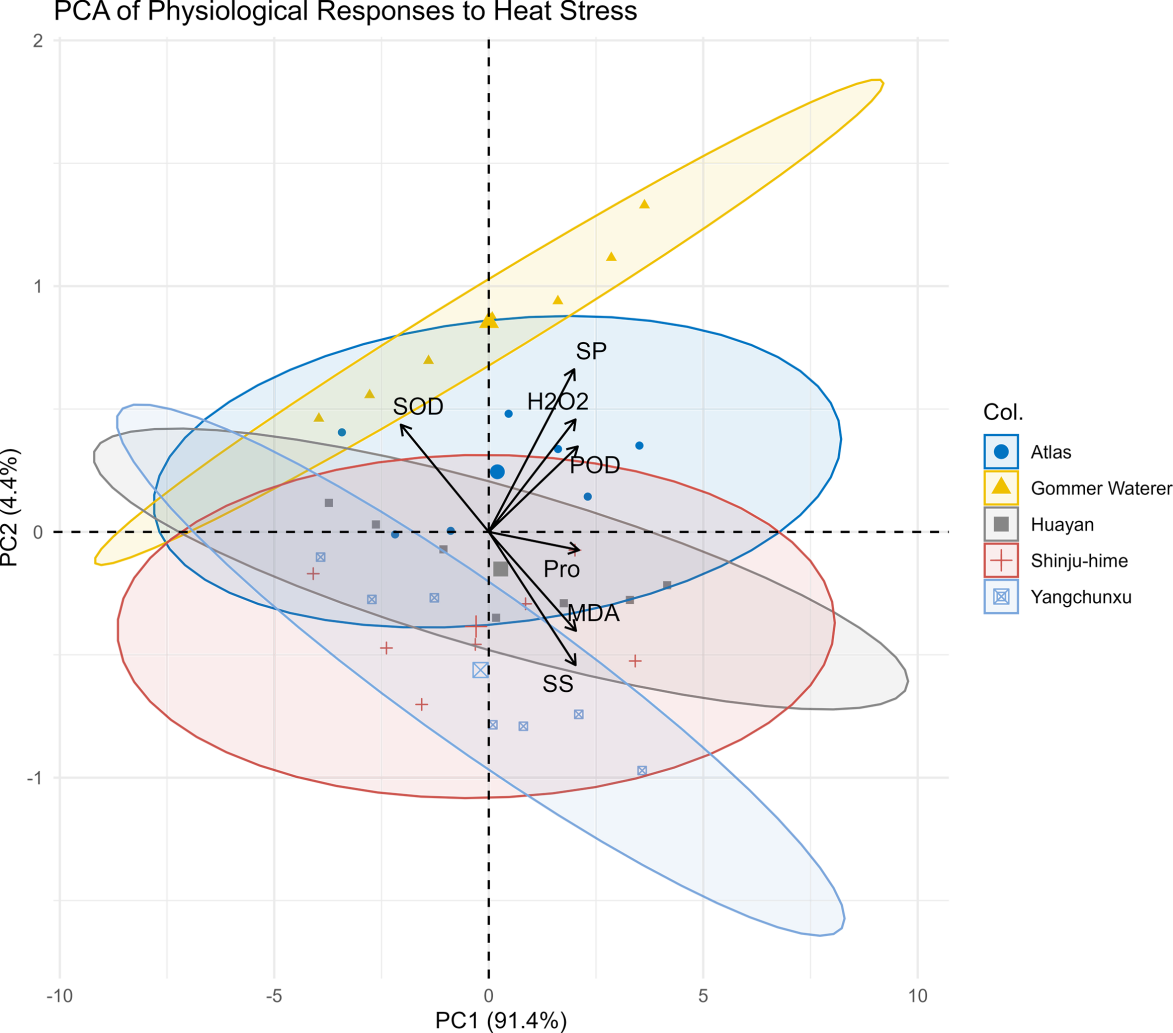
Principal component analysis (PCA) biplot of physiological parameters in five *Rhododendron* cultivars under chronic heat stress. Arrows represent loading vectors of the measured physiological traits: superoxide dismutase (SOD), peroxidase (POD), soluble protein (SP), malondialdehyde (MDA), hydrogen peroxide (H_2_O_2_), proline (Pro), and soluble sugar (SS). Each data point represents a biological replicate at a given time point (0, 5, 10, 20, and 30 d). Shaded ellipses indicate 95% confidence regions for each cultivar across the 30-day treatment period.

A PCA biplot visualized the physiological divergence among the cultivars (**Figure 4**). The first two principal components accounted for 95.8% of the total variance (PC1: 91.4%; PC2: 4.4%). PC1 was strongly associated with variables related to stress severity, exhibiting positive loadings for SP, POD, H_2_O_2_, MDA, Pro, and SS, and a negative correlation with SOD activity. PC2 was associated with SP and SOD. Notably, the spatial distribution of the samples revealed distinct phenotypic clustering. ‘Gommer Waterer’ uniquely occupied the upper-right region of the biplot, showing a strong association with the directional vectors of SP, H_2_O_2_, and POD along PC1, while also exhibiting elevated PC2 scores that aligned with the SOD vector. In contrast, ‘Huayan’ and ‘Shinju-hime’ were positioned in the lower-left region, showing a strong spatial association with the MDA and SS loading vectors. The samples of ‘Atlas’ and ‘Yangchunxue’ were distributed in intermediate positions, with ‘Atlas’ clustering closer to ‘Gommer Waterer’ and ‘Yangchunxue’ overlapping partially with the sensitive cultivars. Based on these spatial clustering patterns and physiological traits, the five cultivars were categorized for subsequent analysis: ‘Gommer Waterer’ as the highly tolerant genotype, ‘Huayan’ and ‘Shinju-hime’ as highly sensitive, and ‘Atlas’ and ‘Yangchunxue’ as intermediate.

### 3.3 Comparative transcriptomic dynamics under chronic HS

#### 3.3.1 Global identification of DEGs and temporal patterns

**Figure 5:**
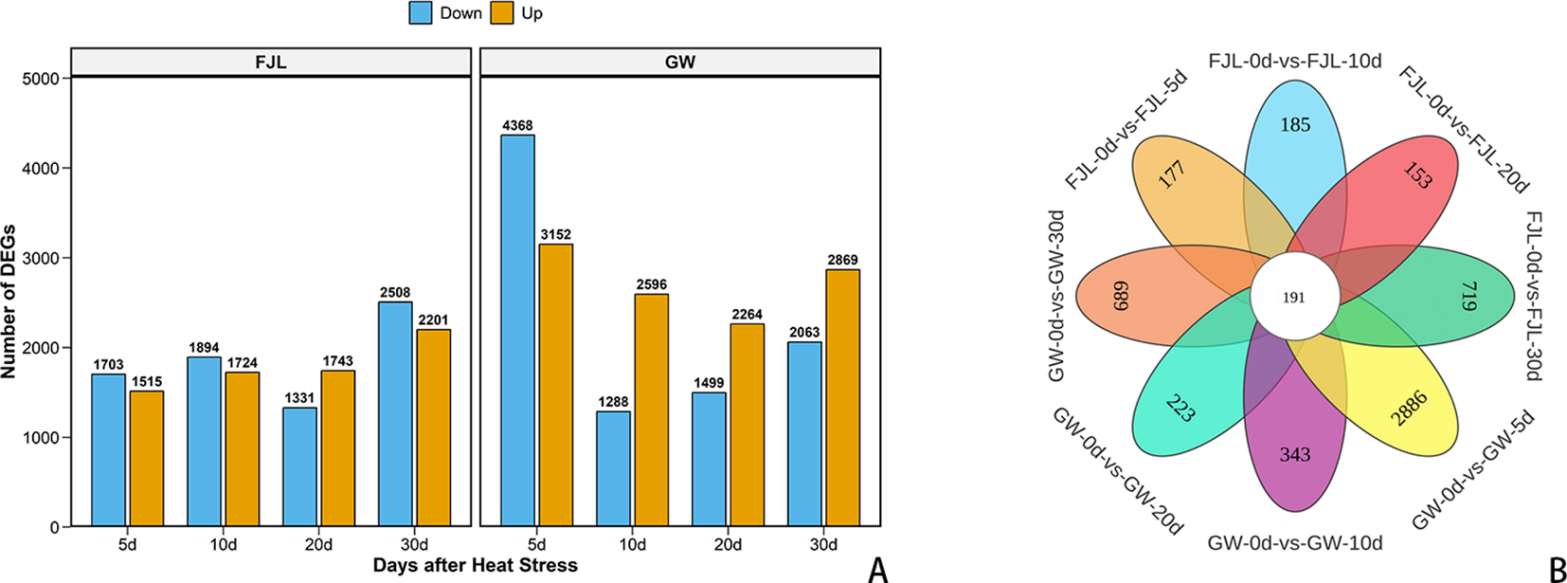
Statistics of differentially expressed genes (DEGs) in *Rhododendron* cultivars ‘GW’ and ‘FJL’. **(A)** Number of up-regulated (orange) and down-regulated (blue) genes at 5, 10, 20, and 30 days of HS compared to the control (0 d). **(B)** Venn diagram illustrating the unique and shared DEGs among different treatment groups.

After normalization, a global analysis of differentially expressed genes (DEGs) was conducted between ‘GW’ and ‘FJL’, which revealed a fundamental divergence in the scale and temporal orchestration of the HS response between the two genotypes (**Figure 5A**).

At the early stage of HS (5 d), ‘GW’ exhibited a rapid and extensive transcriptional mobilization, with a total of 7,520 DEGs identified (3,152 up-regulated and 4,368 down-regulated). This magnitude was more than double the total DEGs identified in ‘FJL’ (3,218) at the same time point. As the chronic stress persisted (20 and 30 d), the total number of DEGs in ‘GW’ gradually decreased and stabilized, although it maintained a robust number of up-regulated genes, consistently staying above 2,000 throughout the entire 30-day period. In stark contrast, the sensitive cultivar ‘FJL’ displayed a delayed transcriptional response, with its DEG count escalating progressively and peaking only at the terminal stage (30 d).

Venn diagram analysis further characterized the specific temporal overlaps of these transcripts (**Figure 5B**). Only a core set of 191 DEGs was continuously shared across all time points and both cultivars. Notably, ‘GW’ possessed the highest number of unique, time-specific DEGs (2,886) at the initial 5 d mark. This substantial accumulation of early-responsive unique transcripts underscores a massive, genotype-specific molecular reprogramming at the onset of stress in the tolerant cultivar.

#### 3.3.2 Functional annotation and pathway enrichment analysis

**Figure 6:**
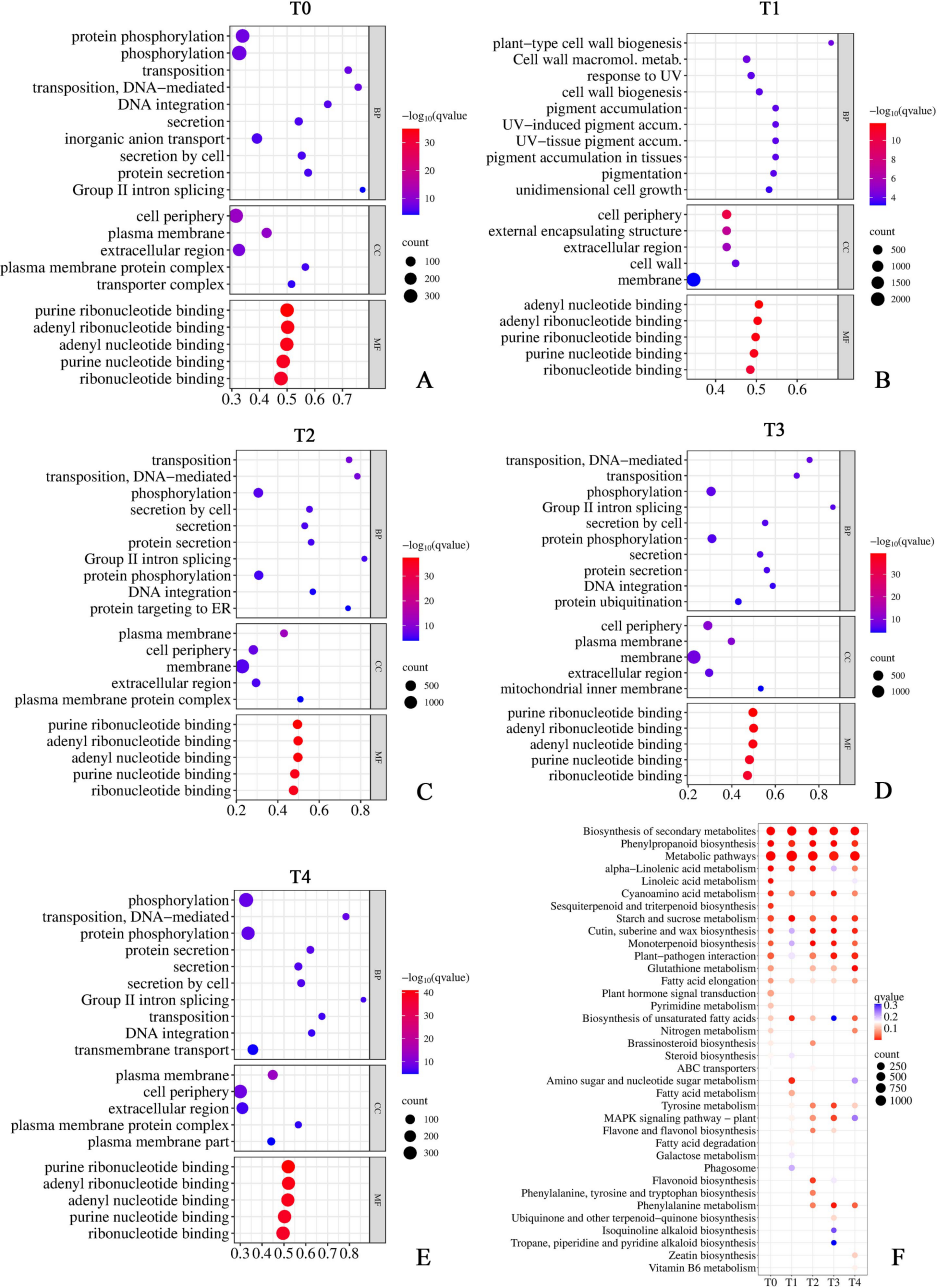
GO and KEGG enrichment analyzes of DEGs between ‘FJL’ and ‘GW’ under chronic HS. **(A–E)** GO enrichment bubble charts at T0–T4, corresponding to FJL_0d vs. GW_0d, FJL_5d vs. GW_5d, FJL_10d vs. GW_10d, FJL_20d vs. GW_20d, and FJL_30d vs. GW_30d, respectively. Bubble size represents gene number, and color indicates −log_10_(q-value). **(F)** KEGG pathway enrichment bubble chart across T0–T4. Bubble size represents gene count, and color indicates q-value. Only pathways with q-value < 0.05 in at least one time point are displayed.

GO and KEGG pathway enrichment analyzes were performed on the DEGs identified from pairwise comparisons between the two cultivars (**Figure 6**). GO enrichment analysis categorized the DEGs into biological process (BP), cellular component (CC), and molecular function (MF) across all five time points (**Figures 6A–E**). In the MF category, nucleotide binding-related terms, including “purine ribonucleotide binding”, “adenyl ribonucleotide binding”, and “ribonucleotide binding”, were consistently the most significantly enriched terms from T0 through T4. In the CC category, “cell periphery”, “plasma membrane”, and “extracellular region” were enriched across all time points. In the BP category, phosphorylation-related terms (e.g., “protein phosphorylation” and “phosphorylation”) were recurrently enriched throughout the stress period. Notably, T1 displayed a distinct BP enrichment profile dominated by cell wall-related terms (e.g., “plant-type cell wall biogenesis” and “cell wall biogenesis”) and pigment accumulation-related terms (e.g., “response to UV” and “pigment accumulation”), which were absent at other time points.

KEGG pathway enrichment analysis revealed that “Biosynthesis of secondary metabolites”, “Phenylpropanoid biosynthesis”, and “Metabolic pathways” were the most consistently and significantly enriched pathways throughout the entire stress period (**Figure 6F**). Other enriched pathways were primarily involved in lipid metabolism, secondary metabolite biosynthesis, and stress signal transduction, with distinct temporal enrichment patterns across T0–T4. Furthermore, “Glutathione metabolism” was enriched at T0 and from T2 to T4, with the highest significance observed at T4. Additionally, lipid-related pathways such as “alpha-Linolenic acid metabolism” and “Cutin, suberine and wax biosynthesis” exhibited specific temporal enrichment patterns during the stress period.

### 3.4 Identification of key gene modules and hub genes via WGCNA

**Figure 7:**
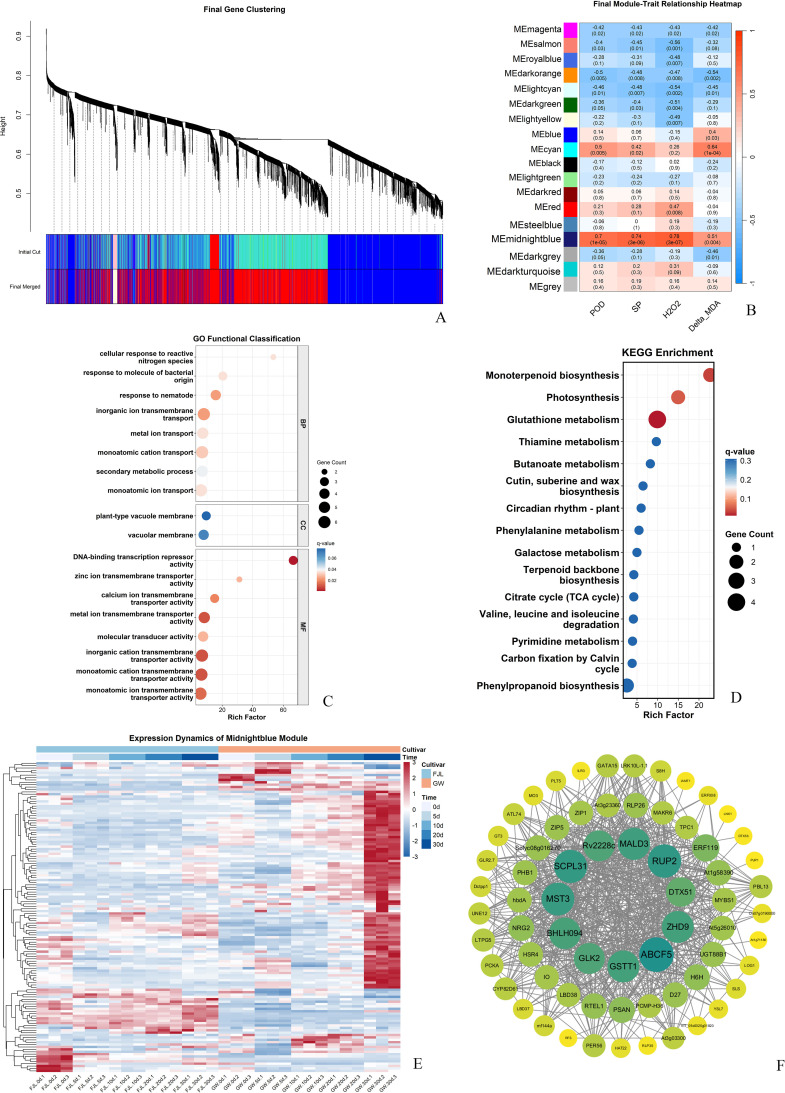
Weighted gene co-expression network analysis (WGCNA) and hub gene identification. **(A)** Hierarchical clustering dendrogram showing the assignment of DEGs into 18 distinct co-expression modules based on topological overlap. **(B)** Module–trait relationship heatmap illustrating Pearson correlation coefficients (with p-values in parentheses) between module eigengenes (MEs) and physiological traits including POD, SP, H_2_O_2_, and normalized MDA (Delta_MDA). **(C)** GO enrichment analysis of genes within the MEmidnightblue module, highlighting key biological processes. **(D)** KEGG pathway enrichment analysis of genes in the MEmidnightblue module. Bubble size represents gene count, and color intensity indicates the significance level (q-value). **(E)** Expression heatmap of genes within the MEmidnightblue module across two *Rhododendron* cultivars (‘FJL’ and ‘GW’) at five heat-stress durations (0, 5, 10, 20, and 30 d). **(F)** Co-expression network visualization of the MEmidnightblue module. Node size is proportional to the connectivity (degree), with core hub genes labeled. The network was constructed using an edge weight threshold > 0.15.

Weighted Gene Co-expression Network Analysis (WGCNA) was performed on 17,340 genes passing variance filtering to identify modules associated with HS. WGCNA identified 18 distinct modules (**Figure 7A**). Among these, the *midnightblue* module (comprising 192 genes) exhibited the strongest positive correlations with all measured physiological traits, including soluble protein (SP; r=0.74, *P* < 0.001), peroxidase (POD; r=0.70, *P* < 0.001), hydrogen peroxide (H_2_O_2_, r=0.78, *P* < 0.001), and Delta_MDA (ΔMDA; r=0.51, *P* < 0.001**) (****Figure 7B**).

To elucidate the functional composition of the *midnightblue* module, GO and KEGG enrichment analyzes were conducted (**Figures 7C, D**). GO analysis showed that genes within this module are primarily involved in “cellular response to reactive nitrogen species” (BP), “plant-type vacuole membrane” (CC), and “DNA-binding transcription repressor activity” (MF). KEGG pathway analysis highlighted a significant enrichment in “Glutathione metabolism”, “Monoterpenoid biosynthesis”, and “Photosynthesis”, suggesting that this module plays a central role in antioxidant detoxification and metabolic reprogramming under HS.

The expression dynamics of the *midnightblue* module genes exhibited a clear genotype-specific pattern (**Figure 7E**). In the heat-sensitive cultivar ‘FJL’, the majority of these genes were repressed or maintained at low basal levels throughout the 30-day heat treatment. In contrast, in the heat-tolerant cultivar ‘GW’, these genes were strongly activated and sustained high expression levels.

To identify the core regulatory nodes, a hub gene network was constructed with an edge weight threshold > 0.15 (**Figure 7F**). Hub genes were identified based on Module Membership (MM) values, with a threshold of MM > 0.90 applied to select candidates (**Supplementary Table 2**). Based on this threshold and connectivity, the top high-confidence hub genes were identified, including *RUP2* (MM = 0.979), *SCPL31* (MM = 0.971), *ZHD9* (MM = 0.970), *ABCF5* (MM = 0.968), and *GSTT1* (MM = 0.967). Notably, *GSTT1* formed co-expression clusters with multiple transcription factors within this module, including *bHLH094*, *ERF119*, and *MYBS1*.

### 3.5 Expression heatmap of the glutathione pathway

**Figure 8:**
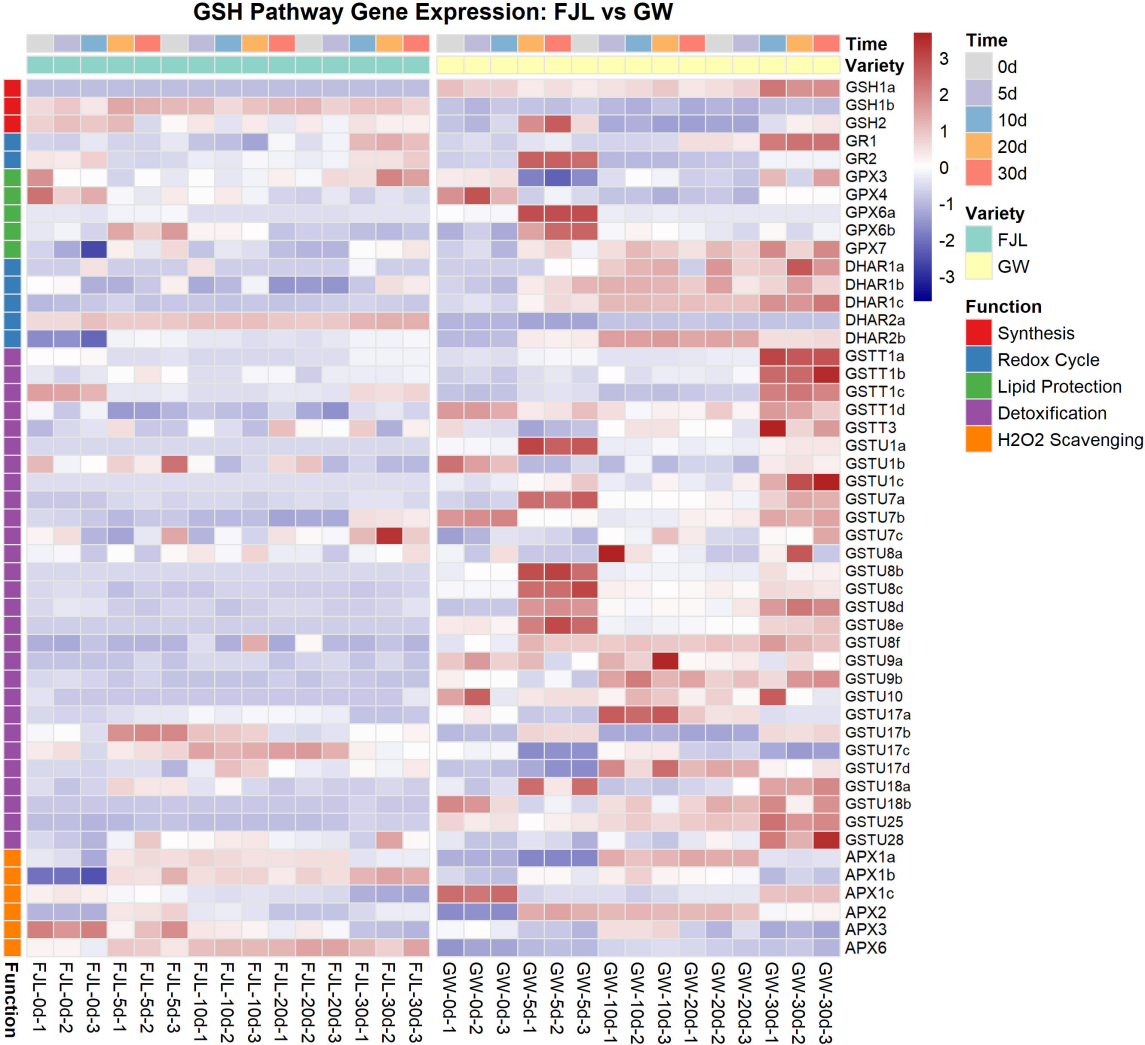
Transcriptional dynamics of the glutathione (GSH) metabolic pathway in *Rhododendron* cultivars under chronic HS. Heatmap illustrating the temporal expression profiles of GSH-related genes in the heat-sensitive ‘FJL’ and heat-tolerant ‘GW’ cultivars across 0, 5, 10, 20, and 30 days of high-temperature treatment. Genes are hierarchically clustered and categorized into five sub-functional modules (indicated by the left color bar): Synthesis (red), Redox Cycle (blue), Lipid Protection (green), Detoxification (purple), and H_2_O_2_ Scavenging (orange). The color scale represents the normalized expression levels (row Z-score of FPKM values), ranging from blue (down-regulation) to red (up-regulation).

The glutathione (GSH) metabolism pathway was selected for in-depth analysis based on the convergence of multiple lines of evidence. First, the midnightblue module exhibited the strongest correlation with physiological indicators of oxidative damage and heat injury. Second, *GSTT1* was identified as one of the top five hub genes within this module based on its high connectivity. Finally, global DEG analysis independently confirmed the consistent enrichment of GSH metabolism across multiple stress durations. To investigate the molecular basis of the observed biochemical changes, the expression profiles of genes involved in the GSH pathway were further analyzed (**Figure 8**). These genes were categorized into five functional categories: Synthesis, Redox Cycle, Lipid Protection, Detoxification, and H_2_O_2_ Scavenging.

The expression heatmap showed divergent transcriptional patterns between the two cultivars. In the heat-sensitive cultivar ‘FJL’, the majority of genes across all GSH sub-modules maintained low expression levels throughout the 30-day treatment (**Figure 8**). In contrast, the heat-tolerant cultivar ‘GW’ exhibited upregulation across the pathway, with temporal shifts observed at different stress stages. During the early phase of HS (5 d), ‘GW’ exhibited higher transcript abundance for genes involved in Synthesis (e.g., *GSH2*), the Redox Cycle (e.g., *GR2*), and Lipid Protection (e.g., *GPX6*). Concurrently, within the Detoxification module, specific *GSTU* family members—notably *GSTU1*, *GSTU7*, and the *GSTU8* cluster—were upregulated.

As the stress progressed to 30 d, ‘GW’ showed increased expression across most functional modules. With the exception of the Scavenging module, almost all genes across the GSH pathway were upregulated. The highest induction at this stage included *DHAR1* (Redox Cycle), the *GSTT1* cluster, and specific *GSTU* members (*GSTU1*, *GSTU7*, and *GSTU25*). Notably, genes in the “H_2_O_2_ Scavenging” module (e.g., the *APX* family) maintained low expression levels in ‘GW’ throughout the stress period (5–30 d) (**Figure 8**).

### 3.6 Validation of transcriptomic data via qRT-PCR

**Figure 9:**
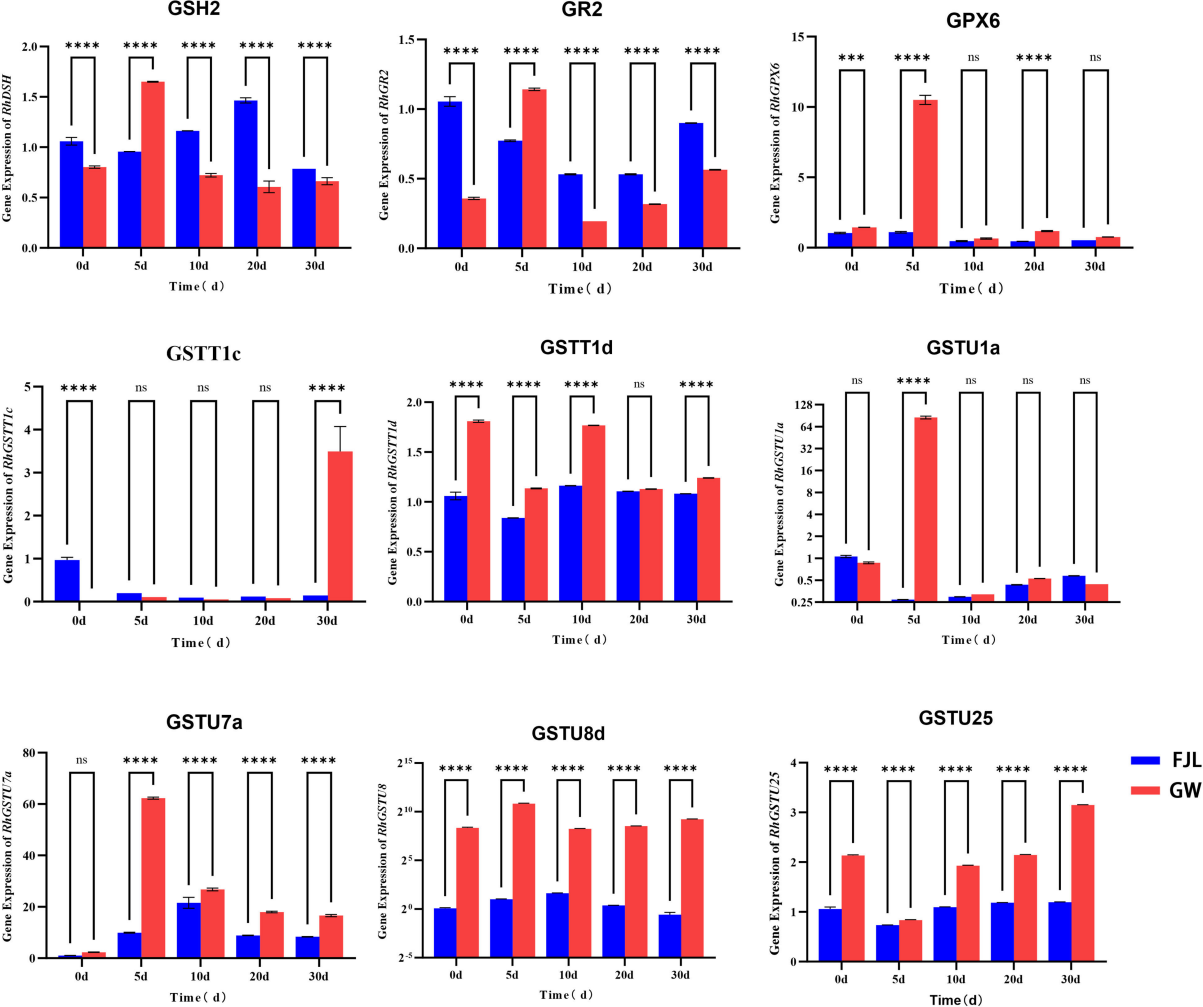
qRT-PCR validation of transcriptomic data for key hub genes in the glutathione (GSH) detoxification network. Relative expression levels of nine representative genes involved in GSH synthesis (*GSH2*), redox cycling (*GR2*), lipid protection (*GPX6*), and ROS detoxification (*GSTT1c*, *GSTT1d*, *GSTU1a*, *GSTU7a*, *GSTU8d*, *GSTU25*) were quantified in the heat-sensitive ‘FJL’ and heat-tolerant ‘GW’ cultivars under chronic HS (0, 5, 10, 20, and 30 d). The *EF-1α* gene was used as the internal reference for normalization. Data are presented as the mean ± standard deviation (SD) of three independent biological replicates. Asterisks indicate statistically significant differences between cultivars at the same time point according to Student’s t-test (*P* < 0.05). Statistical significance: ****, P < 0.0001; ***, P < 0.001; ns, not significant (P ≥ 0.05).

To validate the reliability of the RNA-seq data and the WGCNA findings, nine key genes representing different functional modules of the GSH pathway were selected for quantitative real-time PCR (qRT-PCR) analysis (**Figure 9**). Overall, the relative expression patterns obtained from qRT-PCR were highly consistent with the transcriptomic profiles.

The qRT-PCR results confirmed the genotype-specific temporal activation in the heat-tolerant cultivar ‘GW’. Specifically, genes associated with the early response, including *GSH2*, *GR2*, *GPX6*, *GSTU1a*, and *GSTU7a*, exhibited sharp and significant expression peaks at 5 d in ‘GW’ compared to ‘FJL’ (*P*<0.05). Furthermore, genes involved in robust detoxification, such as *GSTU8d* and *GSTU25*, maintained significantly higher transcript abundance in ‘GW’ than in ‘FJL’ across all chronic stress time points. Notably, the Theta-class GSTs showed distinct survival-related patterns: *GSTT1d* exhibited significantly higher expression in ‘GW’ at multiple stages, particularly reaching a highly significant level at the terminal stage (*P*<0.05), while *GSTT1c* displayed a unique late-phase surge specifically at 30 d. These qRT-PCR results technically confirm the accuracy of the high-throughput sequencing data and reinforce the robust, multi-stage induction pattern of the GSH detoxification network under chronic HS.

### 3.7 Biochemical characterization of the glutathione-mediated antioxidant system

To validate the glutathione detoxification network identified in the transcriptomic analysis, GPX and GST activities and the GSH/GSSG ratio were measured in ‘GW’ and ‘FJL’ under chronic HS.

GPX activity showed distinct temporal dynamics between the two cultivars. At 0 d, basal GPX activity in ‘GW’ was approximately 12-fold higher than that in ‘FJL’. As the treatment progressed, GPX activity in ‘GW’ gradually declined, while ‘FJL’ showed a delayed increase that reached its maximum at 30 d. GST activity in ‘GW’ remained stable during the first 5 d but showed a sharp and significant surge at 10 d (*P*<0.05), markedly exceeding the levels recorded in ‘FJL’ and sustained through 20 d before slightly declining at 30 d. The timing of this enzymatic induction coincided with the transcriptional upregulation of *GSTU* gene clusters observed in the RNA-seq data, confirming the activation of glutathione-mediated detoxification at both the transcriptional and enzymatic levels. ‘FJL’ showed no significant increase in GST activity until 30 d. The GSH/GSSG ratio differed persistently between cultivars throughout the treatment period. ‘FJL’ maintained a consistently higher ratio than ‘GW’ from 5 d to 30 d, peaking at 10 d and remaining elevated thereafter. This pattern in ‘FJL’ coincided with widespread transcriptional repression of the recycling enzymes *GR* and *DHAR* identified in the transcriptomic data, while ‘GW’ maintained a relatively lower but stable ratio throughout the stress period.

## 4 Discussion

This study aimed to elucidate the mechanisms underlying long-term heat adaptation in alpine *Rhododendron*. We hypothesized that chronic heat stress (HS) induces a distinct transcriptional and physiological reconfiguration beyond classical acute heat-shock responses, and that differential survival between cultivars is driven by sustained metabolic homeostasis rather than acute signaling alone. Our results are broadly consistent with this hypothesis: ‘GW’ maintained membrane integrity across the 30-day stress period, accompanied by a rapid and large-scale transcriptional mobilization, with the glutathione (GSH) pathway emerging as a key associated mechanism.

### 4.1 Multi-layered metabolic reprogramming underlies membrane protection in the tolerant cultivar

The substantially larger transcriptional mobilization observed in ‘GW’ at the onset of HS (5 d)—more than double that of ‘FJL’—is consistent with the tolerant cultivar mounting a broader early defense response. This pattern suggests that heat tolerance in *Rhododendron* may involve a multi-layered metabolic reconfiguration rather than a transient stress response, though the precise adaptive significance of each pathway requires further functional investigation.

The consistent enrichment of the phenylpropanoid pathway throughout the stress period is associated with sustained differences in cell wall and secondary metabolite composition between the two cultivars. Lignin deposition and flavonoid accumulation downstream of this pathway have been reported to reinforce structural integrity and provide non-enzymatic antioxidant capacity, which may contribute to limiting lipid peroxidation chain reactions (Ninkuu et al., 2025; Rao et al., 2025). Complementing this, the sustained enrichment of cutin and wax biosynthesis pathways is consistent with enhanced cuticular deposition, associated with reduced transpirational water loss and physical protection under thermal stress (Sonkar and Singh, 2025). Perhaps most informative is the differential regulation of lipid metabolism pathways, specifically alpha-linolenic acid and linoleic acid metabolism. These fatty acids are primary substrates for lipid peroxidation and direct precursors of MDA (Yamauchi et al., 2008); their differential expression between cultivars may reflect distinct membrane lipid remodeling capacities, consistent with lipid remodeling reported in heat-stressed *Arabidopsis* (Higashi et al., 2018), and may contribute to the comparatively lower MDA accumulation observed in ‘GW’. Collectively, these transcriptomic patterns suggest that the metabolic divergence between the two cultivars is associated with differential investment in membrane and structural protection under chronic HS.

Alongside these transcriptomic differences, a physiologically notable pattern emerged: ‘GW’ exhibited the highest H_2_O_2_ concentrations among all cultivars by 30 d, yet maintained comparatively stable MDA levels throughout the stress period (**Figures 3D, E**). This dissociation can be understood in the context of the lipid peroxidation pathway: MDA is generated downstream of lipid hydroperoxides (LOOH), which are the primary peroxidation intermediates and the direct substrates of GPX and GST enzymes (Hasanuzzaman et al., 2017; Yamauchi et al., 2008). Therefore, GST/GPX-mediated removal of LOOH can limit MDA accumulation independently of upstream H_2_O_2_ levels. Consistent with this, the *APX* family—a primary H_2_O_2_ scavenging gene family—showed consistently low expression in ‘GW’ (**Figure 8**), while GSH-dependent detoxification genes were broadly upregulated, suggesting that H_2_O_2_ is less actively scavenged while downstream lipid peroxidation products are preferentially targeted for removal (Hasanuzzaman et al., 2020, Hasanuzzaman et al., 2017). Whether H_2_O_2_ functions as an active signaling molecule in this context, as reported in heat stress systems involving HSF–HSP cascade regulation (Guan et al., 2013), remains to be directly tested. Together, these observations suggest that ‘GW’ shows a pattern more consistent with targeted lipid peroxidation detoxification rather than broad-spectrum ROS scavenging.

### 4.2 Convergent biochemical and network evidence highlights the GSH pathway

**Figure 10:**
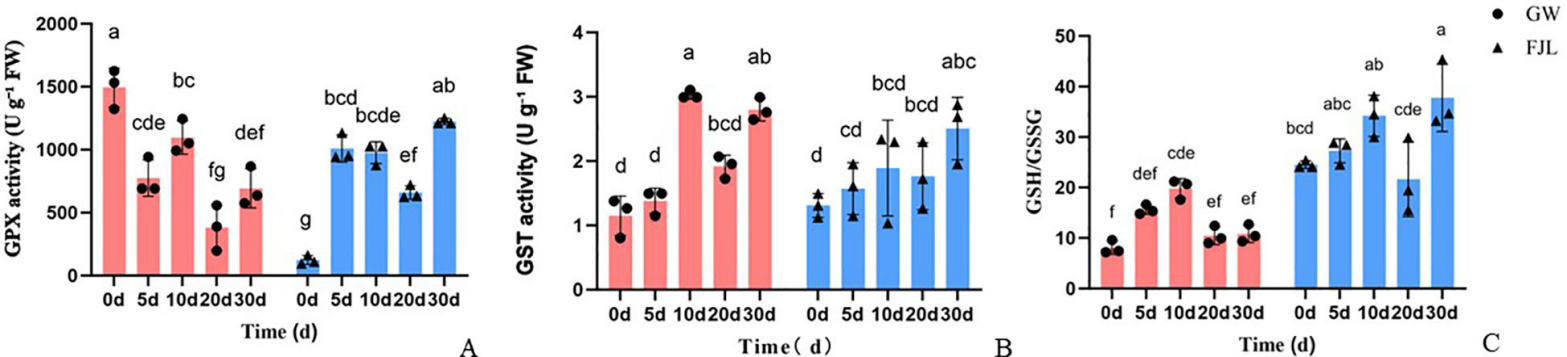
Effects of chronic heat stress on the glutathione-mediated antioxidant system in leaves of the thermotolerant (‘GW’) and heat-sensitive (‘FJL’) *Rhododendron* cultivars. **(A)** Glutathione peroxidase (GPX) activity. **(B)** Glutathione S-transferase (GST) activity. **(C)** The ratio of reduced to oxidized glutathione (GSH/GSSG). The plants were subjected to chronic heat stress for 0, 5, 10, 20, and 30 days. Data are presented as the mean ± standard deviation (SD) of three biological replicates (n = 3). Different lowercase letters above the bars indicate statistically significant differences across all treatments and cultivars, according to Tukey’s multiple comparison test (*P* < 0.05).

The identification of the *midnightblue* module by WGCNA, with “Glutathione metabolism” among its most enriched pathways (**Figure 7D**), provided a network-level basis for linking transcriptomic reprogramming to the physiological divergence observed between cultivars. Biochemical characterization of the GSH system provided convergent support for this network finding at three levels. First, ‘GW’ exhibited a markedly higher basal GPX activity at 0 d (approximately 12-fold higher than ‘FJL’; **Figure 10A**), suggesting that the tolerant cultivar possesses a substantially higher constitutive capacity for glutathione-dependent peroxide metabolism prior to stress onset. Second, as HS progressed, GST activity in ‘GW’ showed a significant and sustained surge from 10 d onwards (**Figure 10B**), temporally coinciding with the transcriptional upregulation of *GSTU* gene clusters and the hub gene *GSTT1* observed in the RNA-seq data (**Figure 8**), suggesting that the enzymatic surge in ‘GW’ is consistent with transcriptional activation of the GSH detoxification network. Third, the GSH/GSSG ratio revealed a contrasting pattern between the two cultivars. ‘FJL’ maintained a consistently higher GSH/GSSG ratio from 5 d to 30 d, which might superficially indicate a stronger reducing environment; however, this was accompanied by widespread transcriptional repression of the recycling enzymes *GR* and *DHAR* (**Figure 8**). Since GR is the primary enzyme responsible for regenerating GSH from GSSG in an NADPH-dependent reaction, and DHAR links the ascorbate and glutathione pools during H_2_O_2_ detoxification, impaired expression of both enzymes suggests that the elevated GSH/GSSG ratio in ‘FJL’ more likely reflects reduced GSH consumption and impaired redox cycling rather than active antioxidant defense—a pattern consistent with metabolic stagnation under prolonged oxidative stress (Dorion et al., 2021; Hasanuzzaman et al., 2017). In contrast, ‘GW’ maintained a comparatively lower but stable GSH/GSSG ratio throughout the treatment period, a pattern consistent with active and continuous GSH turnover in which GSH is consumed by detoxifying enzymes and regenerated through the GR-mediated recycling pathway, rather than accumulating passively (Dorion et al., 2021). Collectively, these three lines of biochemical evidence are consistent with the interpretation that ‘GW’ maintains a more functionally active GSH system under chronic HS, characterized by higher enzymatic throughput and dynamic redox cycling, rather than passive glutathione accumulation.

The involvement of GSH in heat stress tolerance is well-established in the literature. Overexpression of a bifunctional GST/GPX gene in tobacco reduced lipid hydroperoxide accumulation and maintained seedling growth under heat stress, establishing that GST-mediated detoxification is a key mechanism limiting oxidative membrane damage (Roxas et al., 2000). In *Arabidopsis*, overexpression of the pepper transcription factor *CaHsfA1d* enhanced thermotolerance by specifically upregulating the tau-class *AtGSTU5* and *AtGPX3*, while classic antioxidant enzyme genes (*APX*, *CAT*, *SOD*) showed no significant induction (Gai et al., 2020), directly demonstrating that GST-mediated detoxification, rather than broad-spectrum ROS scavenging, is a functionally significant component of heat tolerance. Critically, *Arabidopsis* glutathione-deficient mutants (*cad2* and *pad2*) exhibited markedly reduced survival under high temperature, confirming that GSH availability is a prerequisite for thermotolerance (Dard et al., 2023). Notably, within the *Rhododendron* genus itself, transcriptomic and metabolomic analysis of *R. henanense* subsp. *lingbaoense* under heat stress revealed broad upregulation of GST-encoding genes alongside suppression of HSP-related genes (Li et al., 2025), suggesting that GST-mediated detoxification may represent a conserved heat response strategy in thermally challenged *Rhododendron* species. The high connectivity of *GSTT1* in the tolerant network—and its co-expression with transcription factors *bHLH094* and *ERF119*—marks it as a strong candidate for further functional validation. To our knowledge, the involvement of Theta-class GSTs in plant heat tolerance has not been previously reported, identifying *GSTT1* as a candidate for future functional investigation.

### 4.3 Study limitations and future perspectives

Several limitations of the present study warrant consideration. First, the present study is observational in design: the associations identified between GSH pathway gene expression, *GSTT1* network connectivity, and membrane stability are correlational, and do not constitute direct evidence of causality. Functional characterization of *GSTT1* in future studies would be required to establish its potential causal role in thermotolerance. Second, the chronic heat stress treatments were conducted under controlled environmental chamber conditions designed to simulate the diurnal temperature profiles of subtropical summers; however, such conditions necessarily simplify the complexity of natural field environments, where additional abiotic factors including soil moisture fluctuation, UV radiation, and variable humidity may interact with heat stress to modify plant responses. Future work should therefore seek to validate the key findings reported here under field conditions and across a wider panel of *Rhododendron* cultivars, with the ultimate goal of translating these candidate mechanisms into practical heat-tolerance breeding strategies.

## 5 Conclusion

This study provides convergent evidence suggesting that the heat-tolerant *Rhododendron* cultivar ‘Gommer Waterer’ maintains membrane stability under chronic heat stress despite elevated H_2_O_2_ accumulation. Integrated physiological and transcriptomic analyzes revealed extensive metabolic reprogramming in the phenylpropanoid, lipid, and glutathione pathways. WGCNA identified the midnightblue co-expression module as significantly correlated with heat resilience traits and enriched in glutathione metabolism, within which *GSTT1*—a Theta-class glutathione S-transferase—was identified as a highly connected hub gene. Biochemical assays corroborated these findings, with ‘GW’ showing higher constitutive GPX activity, a stress-induced surge in GST activity, and dynamic GSH/GSSG cycling, collectively supporting glutathione-mediated detoxification at both the transcriptional and enzymatic levels. These findings provide new insights into chronic heat adaptation mechanisms in woody ornamentals and identify candidate targets for future functional validation and heat-tolerance breeding strategies.

## Data Availability

The data presented in the study are deposited in the NCBI SRA repository, accession numbers: BioProject, PRJNA1464444; SRA Study: SRP699886; SRA Run accessions: SRR38556183–SRR38556211. Further inquiries can be directed to the corresponding authors.
